# Is Homeopathic *Arnica* Effective for Postoperative Recovery? A Meta-analysis of Placebo-Controlled and Active Comparator Trials

**DOI:** 10.3389/fsurg.2021.680930

**Published:** 2021-12-17

**Authors:** Katharina Gaertner, Stephan Baumgartner, Harald Walach

**Affiliations:** ^1^Institute for Integrative Medicine, University of Witten/Herdecke, Herdecke, Germany; ^2^Institute of Complementary and Integrative Medicine, University of Bern, Bern, Switzerland; ^3^Society for Cancer Research, Arlesheim, Switzerland; ^4^Change Health Science Institute, Berlin, Germany

**Keywords:** *Arnica montana*, wound healing, pain, homeopathy, meta-analysis

## Abstract

**Background:** Homeopathic *Arnica montana* is used in surgery as prevention or treatment for the reduction of pain and other sequelae of surgery. Our aim was to perform a metaanalysis of clinical trials to assess efficacy of *Arnica montana* to reduce the inflammatory response after surgery.

**Method:** We conducted a systematic review and metaanalysis, following a predefined protocol, of all studies on the use of homeopathic *Arnica montana* in surgery. We included all randomized and nonrandomized studies comparing homeopathic *Arnica* to a placebo or to another active comparator and calculated two quantitative metaanalyses and appropriate sensitivity analyses. We used “Hegde's g,” an effect size estimator which is equivalent to a standardized mean difference corrected for small sample bias. The PROSPERO registration number is CRD42020131300.

**Results:** Twenty-three publications reported on 29 different comparisons. One study had to be excluded because no data could be extracted, leaving 28 comparisons. Eighteen comparisons used placebo, nine comparisons an active control, and in one case *Arnica* was compared to no treatment. The metaanalysis of the placebo-controlled trials yielded an overall effect size of Hedge's g = 0.18 (95% confidence interval −0.007/0.373; *p* = 0.059). Active comparator trials yielded a highly heterogeneous significant effect size of g = 0.26. This is mainly due to the large effect size of nonrandomized studies, which converges against zero in the randomized trials.

**Conclusion:** Homeopathic *Arnica* has a small effect size over and against placebo in preventing excessive hematoma and other sequelae of surgeries. The effect is comparable to that of anti-inflammatory substances.

## Introduction

Homeopathic *Arnica montana* has a reputation for stemming bleeding, mainly internal bleeding following internal lacerations, for instance from contusions, concussions, sprains, and other injuries (1–5). Hahnemann, the founder of Homeopathy, took this indication from European folk-medicine, where *Arnica* has been long in use for the treatment of sprains and lacerations. The principle of homeopathy is the law of similars, *similia similibus curentur*, let like be cured by like (6). Hahnemann developed an operationalization by using known toxicological data from poisonings with various plants and substances, and the symptoms that these substances produce when ingested by healthy volunteers, either crudely, or more often in potentized form (7). For this purpose, the substances are successively diluted and succussed (i.e.. vigorously shaken) in a series of dilutions, and the probability to find any molecule of the potentized substance rapidly converges to zero for homeopathic potencies beyond 24X or 12C. Hence, it is unclear how such potencies can at all be effective. However, reviews of pathogenetic trials in healthy volunteers document effects that are different from placebo, at least sometimes (8), and a recent meta-analysis shows that individualized homeopathy can be statistically separated from placebo (9). The bone of contention is of course the question as to how remedies without a known active ingredient could be at all effective. We plead ignorance here. But absence of knowledge is not knowledge of absence. It might well be possible, as has been often the case in medicine, that future theories or further research might unearth some new therapeutic principle. To understand whether this is at all a useful exercise, it is helpful to see whether the data to date would justify such efforts.

*Arnica* is used a lot for self-care (10, 11) and also in midwifery (12) and surgery (13). Meanwhile, some 30 studies have been conducted where homeopathic *Arnica* has been applied before or after surgery to improve wound healing, stop bleeding and swelling, and reduce pain. Studies of *Arnica* for the prevention of muscle-soreness after sportive activities such as marathon running are negative (14, 15), and an early systematic review was skeptical about the effectiveness of *Arnica* (16). A recent publication summarized the effects of *Arnica* as found in 20 placebo-controlled trials descriptively, and the authors stated that homeopathic *Arnica* “seems to have a mitigating effect on ecchymosis, most notably following rhinoplasty and facelifts/facial procedures” and claimed the importance to determine the efficacy of this possibly beneficial therapeutic intervention (17).

Hence, we set out to conduct a systematic review and meta-analysis of clinical trials asking whether homeopathically prepared *Arnica* is more effective than placebo, or at least as effective as active controls in surgery for the treatment of postoperative pain, prevention of swelling, edema, and other sequelae. We deliberately focused on studies that used *Arnica* only and excluded trials that used a combination of remedies.

## Method

We developed a protocol that was logged with the PROSPERO database before the commencement of the study (Registration number: CRD42020131300; available from https://www.crd.york.ac.uk/prospero/display_record.php?ID=CRD42020131300). One reviewer conducted the search using this predefined search strategy. We included all trials that produced a randomized or quasi-randomized comparison between homeopathic *Arnica* in any potency and dosage, and placebo or an active control in surgical procedures. Arnica tinctures without homeopathic processing into potencies were excluded. All references between January 1, 1980 and January 25, 2020 were eligible for screening. There were no language restrictions.

**Figure 1:**
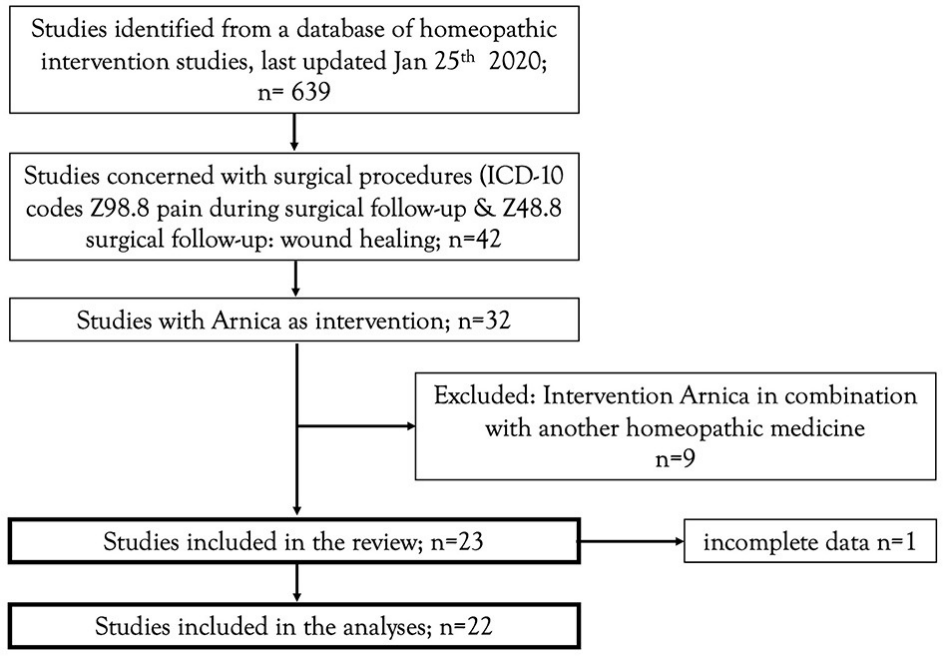
Flowchart of studies included in the review.

The present project was carried out as part of a general update of empirical evidence of homeopathic interventions. The search strategy of this general project aimed to identify controlled clinical investigations (randomized or non-randomized), employing one or more homeopathically processed substances on humans exhibiting a clinically relevant disease (treatment interventions) or on humans in danger of developing a disease (prophylactic or preventive interventions). The complete search strategy and classification methods of the literature overview is available in the framework-protocol of the project (18). The surgical studies eligible for this review were deducted from the literature overview. The deduction rules are available as a Supplementary Material (eligible studies). The flowchart for the eligible studies is represented in Figure 1.

The information was extracted into a spreadsheet mirroring the predefined variables by one reviewer (HW) and checked 100% by a second reviewer (KG). The extraction process was documented in a log (Supplementary Material 2), with all changes, conversions, and procedures. Discrepancies between the reviewers were discussed, the results logged, and the database adapted and saved under a new name, such that all changes can be followed.

We extracted the following formal information: publication type, time and country, type of homeopathy (preventive, individualized, or formulaic), type of dilution, diagnoses (ICD10 code), and also quantitative information such as: mean values and standard deviations of outcome criteria. Where a primary outcome was defined, we extracted the primary outcome. Very often multiple outcomes were stipulated and no primary outcome was mentioned. In that case all outcome-variables were extracted. If multiple outcomes were mentioned, the protocol stipulated that each outcome is converted into an effect size and averaged within a study, such that each study entered the analysis with one effect size. Sometimes studies had multiple arms (for instance *Arnica* vs. placebo vs. anti-inflammatory substance). Such studies were coded as two separate studies, one homeopathy vs. placebo and the other one as homeopathy vs. conventional treatment. Two analyses were calculated, one for studies that compared *Arnica* with placebo, and one for those that used a standard-treatment comparator.

We used two quality assessments, the Cochrane risk of bias tool (Version 1.0) and the Quality Assessment Tool for Quantitative Studies, developed by the Effective Public Health Practice Project (EPHPP) (19, 20). This latter summary coding was used as a moderator in the analysis.

Where data were only available in graphs, data were read off the graph by enlarging the display and reading the figures with a ruler. When only partial information was given, we converted the available information, for instance we used published formulae for converting median data into mean (21), or odds ratios (22). Sometimes data were given as categories. These were converted into quasi-continuous data such that mean and standard deviations could be calculated. As the majority of the studies used continuous outcome measures, we decided to use Hedge's g as the effect size measure because it corrects for the typically small study sizes in such trials. If decisions had to be made, they were all documented in the log and the more conservative option was adopted at all times. If only standard errors of the mean were given, or statistical results, such as *p*-values, the necessary data were derived from the standard statistical formulae. As a denominator to calculate Hedge's g, the pooled standard-deviation was used.

The statistical model was defined beforehand as a fixed model, if heterogeneity should turn out to be small. But we expected high heterogeneity from the outset and thus opted for a random effects model. Thus the analytical logic followed the rule “calculate a fixed effects model, if heterogeneity is high calculate a random effects model that incorporates heterogeneity, and conduct sensitivity analyses to study the source of heterogeneity” (1, 23). All calculations were done with comprehensive meta-analysis (24).

## Results

Our search strategy yielded 23 separate publications (25–47) which produced 29 distinct comparisons. One study was so badly documented (26) that no data could be extracted and was rejected, leaving 22 publications and 28 comparisons. In nine comparisons active controls were used, and anti-inflammatory drugs were used in all but one case, where Arnica was compared to Metronidazole, and in one case Arnica was compared to no treatment. Eighteen placebo-controlled trials were found. Three of the active comparator studies were controlled clinical trials, i.e. it was unclear whether they are randomized or a pseudo-randomization procedure, such as alternating patients, was used. One study was nonrandomized and patients could choose the treatment, all others (*n* = 24) were randomized trials. Descriptions of the studies are presented in Table 1 (included studies) and Supplementary Table (excluded studies: Supplementary Material 3).

**Table 1:** Characteristics of included studies.

**ICD-10 category**	**Author**	**Year**	**Condition/ pathology**	**Publi-cation status**	**Study design**	**Intervention/ Comparators**	**Sample Size (ITT (I/C))**	**Lost to FU (I/C)**	**Outcomes**	**Risk-of-bias/ quality**
**Preventive use**										
Z48.8 surgical follow up: wound healing	Brinkhaus et al. (25)	2006	Arthroscopy	peer-review	RCT	*Arnica* 30X/ Placebo	227 (116/111)	1/3	knee circumference, pain, analgesics taken, frequency and quantity of drainage, unexpected events	Low
			Artificial knee replacement				35 (16/19)	1/0		
			Cruciate ligament				57 (30/27)	6/3		
	Chaiet et al. (27)	2016	Rhinoplasty Surgery	peer-review	RCT	*Arnica* 12C+M/ Placebo	26 (12/14)	3/1	extent of ecchymosis	Low^1^
	Del Puerto Horta and Cañete Villafranca (38)	2015	Dental extractions	non peer-review	NRS	*Arnica* 30C/ Metamizol	80 (40/40)	0/0	evolution of pain, adverse events	Weak^10^
	Erkan et al. (28)	2019	Dental surgeries	peer-review	RCT	*Arnica* 200C/ Placebo	94 (47/47)	5/2	patients assessment of pain, swelling, sleeping, eating, phonetics, daily routine & surgeons assessment of operation	Low^1^
	Hart et al. (30)	1997	Hyster-ectomy	peer-review	RCT	*Arnica* 30C/ Placebo	93 (47/46)	9/11	infection rate, analgesics taken, pain scores	Moderate^2^
	Kaziro (33)	1984	Dental extractions	peer-review	RCT	*Arnica* 200C/ Metronidazol Placebo/	80 (39/41)/ 77 (39/38)	0/0	pain scores, trismus, edema, wound healing	High^3^
	Kotlus et al. (34)	2010	Eyelid surgery	peer-review	RCT	*Arnica* 12C+M/ Placebo	30 (60 eyes)	3	extent of ecchymosis, patients assessment of success	Low
	Macedo et al. (35)	2005	Dental extractions	non peer-review	NRS	*Arnica* 6C/ Placebo	32 (64 teeth)	0	edema, mouth opening, pain, demand of analgesics	Moderate^4^
	Pinsent et al. (36)	1986	Dental extractions	non peer-review	RCT	*Arnica* 30C/ Placebo	100 (50/50)	7/4	pain-, bleeding- & severity score	Moderate^5^
	Pöllmann (37)	1985	Dental extractions	non peer-review	NRS	*Arnica* 3X/ Bromelaine 100U/ Paracetamol 250mg on demand	25 (18/7)/ 36 (18/18)	0/0	edema, mouth opening, demand of analgesics	Weak^10^
	Ramelet et al. (39)	2000	Venous stripping	peer-review	RCT	*Arnica* 5C/ Placebo	130 (65/65)	0/0	extent of haematoma	Low^6^
	Seeley et al. (41)	2006	Face lifts	peer-review	RCT	*Arnica* 12C+M/ Placebo	29 (14/15)	0/3	extent of haematoma	Low
	Sorrentino et al. (42)	2017	Mastectomy in Breast Cancer	non peer-review	RCT	*Arnica* M/ Placebo	53 (26/27)	3/7	drainage volume	Low
	Souza (43)	2011	Dental extractions	peer-review	RCT	*Arnica* 6C/ Diclofenac 50mg	30 (60 teeth)	0	swelling	Moderate^7^
	Stevinson et al. (44)	2003	Carpal tunnel surgery	peer-review	RCT	*Arnica* 6C/ Arnica 30C/ Placebo	43 (21/22)/ 43 (21/22)	1/0	pain scores, haematoma, swelling, analgesics taken	Low
	Wolf and Rose (46)	2002	Surgical treatment of hip fractures	non peer-review	NRS	*Arnica* 12X/ standard care	40 (20/20)	0/0	thigh circumference	Weak^10^
	Wolf et al. (47)	2003	Venous stripping	peer-review	RCT	*Arnica* 12X/ Placebo	59 (30/29)	0/0	extent of haematoma, pain scores	Low
**Therapeutic use**										
Z48.8 surgical follow up: haematoma & edema	González Sánchez et al. (29)	2014	Strabism surgery	non peer-review	RCT	*Arnica* (unknow potency)/ Prednisolone eye drops	100 (50/50)	0/0	grade of quemosis and bleeding	High^8^
	Totonchi and Guyuron (45)	2007	Rhino-plasty Surgery	peer-review	RCT	*Arnica* 12C+M/ Cortisone i.v.+orally/ no treatment	32 (16/16)/ 32 (16/16)	0/0	extent of ecchymosis	High^9^
Z98.8 Pain during surgical Follow-up	Jeffrey et al. (31)	2002	Carpal tunnel surgery	peer-review	RCT	*Arnica* 6X/ Placebo	37 (20/17)	0/0	wrist circumference, grip strength	Low^6^
	Karow et al. (32)	2008	Halux valgus surgery	peer-review	RCT	*Arnica* 4X/ Diclofenac 50mg	88 (44/44)	0/0	postoperative irrtation (rubor, swelling, calor), convalescence, pain scores, analgesics taken	Low^6^
	Robertson et al. (40)	2007	Tonsil-ectomy	peer-review	RCT	*Arnica* 30C/ Placebo	190 (93/97)	40/39	pain scores, analgesics taken, complications, return of normal swallowing and to work	Low^1^

*Description of included 22 publications reporting on 28 comparisons: C, Control; FU, Follow-up; I, Intervention; NRS, non randomized study; RCT, randomized controlled trial; ^1^Unclear risk of bias for the domain attrition; ^2^Unclear risk of bias for the domains attrition and reporting; ^3^Unclear risk of bias for the domains allocation concealment, blinding & outcome assessment; ^4^Assessed with the quality-assessment tool for quantitative studies by Thomas et al. (19): weak for the domain data collection methods; ^5^Unclear risk of bias for the domains randomization, allocation, blinding & reporting; ^6^Unclear risk of bias for the domain allocation concealment; ^7^No blinding; ^8^Unclear risk of bias for the domains randomization and allocation concealment, no blinding; ^9^Unclear risk of bias for the domain allocation concealment and no blinding; ^10^Assessed with the quality-assessment tool for quantitative studies by Thomas et al. (19): poor quality for at least two domains*.

### Arnica vs. Placebo

**Figure 2:**
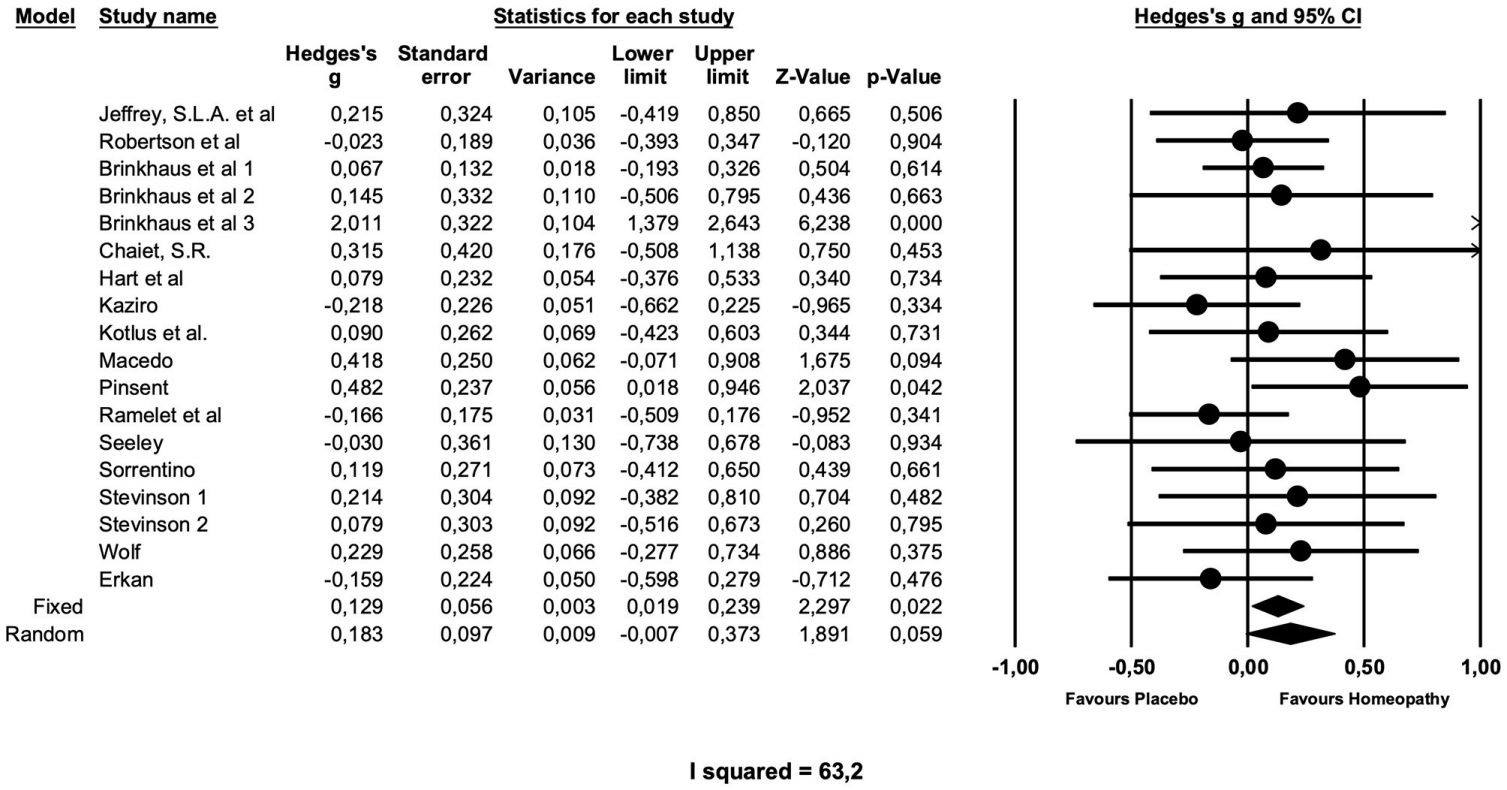
Forest plot of main meta-analysis of placebo-controlled trials of *arnica* in postoperative complications.

Table 2 gives the results of the main meta-analyses and sensitivity analyses, and Figure 2 presents the main analysis as forest plot. The overall analysis of all comparisons with placebo yielded a small effect size just missing formal significance of Hedge's *g* = 0.18 (*z* = 1.89; *p* = 0.059), which was chosen, because the fixed effects model produced a very heterogeneous summary (*I*^2^ = 63.2; *g* = 0,13; *z* = 2.29; *p* = 0.02).

**Table 2:** Summary of meta-analysis.

**Analysis**	**Model**	**Hedge's g**	**Heterogeneity I^**2**^**	**z-score**	**p-value**	**95% CI of g**
**Placebo-controlled studies**						
**Overall**	fixed k = 18	0.13	63.2	2.29	0.02	0.02/0.24
“	*random* k = 18	*0.18*	–	*1.89*	*0.059*	*−0.01/0.37*
**Sensitivity 1** Preventive use	fixed k = 16	0.14	67.0	2.40	0.018	0.02/0.26
	*random* *k = 16*	*0.20*	–	*1.90*	*0.06*	*−0.01/0.4*
Therapeutic use	*fixed* *k = 2*	*0.04*	*0*	*0.20*	*0.80*	*−0.30/0.36*
**Sensitivity 2** Potency high	fixed k = 17	0.13	65.3	2.21	0.027	0.01/0.24
**“**	*random* k = 17	*0.18*	–	*1.81*	*0.07*	*−0.02/0.38*
Potency low	*fixed* k = 1	*0.21*	*0*	*0.66*	*0.5*	*−0.41/0.85*
**Sensitivity 3** Study quality low or moderate	*fixed* k = 10	*0.09*	*0*	*1.07*	*0.5*	*−0.07/0.24*
Study quality high	fixed k = 8	0.17	81.1	2.15	0.03	0.01/0.33
“	*random* k = 8	*0.29*	–	*1.90*	*0.057*	*−0.01/0.58*
**Other-than-placebo-controlled Trials**						
**Overall**	fixed k = 10	0.26	78.1	3.1	0.002	0.1/0.42
	*random* *k = 10*	*0.28*	–	*1.52*	*0.13*	–*0.08/0.64*
**Sensitivity 4** Active comparator studies	fixed k = 9	0.26	80.5	3.0	0.003	0.09/0.43
	*random* *k = 9*	*0.28*	–	*1.40*	*0.16*	–*0.11/0.67*
**Sensitivity 5** Compared against standard care	*fixed* k = 2	*0.50*	*0*	*2.16*	*0.03*	*0.05/0.96*
**Sensitivity 6** Non-randomized studies	fixed k = 6	0.49	7.0	4.50	<0.001	0.28/0.70
	*random* k = 6	*0.49*	–	*2.30*	*0.02*	*0.08/0.90*
Randomized	fixed k = 4	−0.10	75.3	−0.70	0.4	−0.40/0.16
	*random* k = 4	*−0.03*	–	*−0.10*	*0.9*	*−0.60/0.50*
**Sensitivity 7** Preventive use	fixed k = 6	0.16	77.2	1.40	0.15	−0.06/0.40
	*random* *k = 6*	*0.23*	–	*0.40*	*0.9*	*−0.30/0.72*
Therapeutic use	fixed k = 4	0.38	82.9	3.00	0.002	0.13/0.62
	*random* *k = 4*	*0.35*	–	*1.20*	*0.2*	*−0.30/0.70*
**Sensitivity 8** Study quality high	fixed k = 3	0.45	88.1	3.30	0.001	0.18/0.71
	*random* *k = 3*	*0.44*	–	*1.30*	*0.2*	*−0.22/1.11*
Study quality low	fixed k = 7	0.15	71.9	1.40	0.17	−0.06/0.40
	*random* k = 7	*0.21*	–	*0.90*	*0.4*	*−0.20/0.60*
**Sensitivity 9** Potency high	fixed k = 6	0.12	75.1	1.10	0.3	−0.09/0.33
	*random* *k = 6*	*0.15*	–	*0.72*	*0.5*	*−0.26/0.57*
Potency low	fixed k = 3	0.10	51.4	0.58	0.6	−0.23/0.42
	*random* *k = 3*	*0.22*	–	*0.71*	*0.5*	*−0.39/0.48*
Potency unknown	fixed k = 1	1.1	–	5.0	<0.001	0.65/1.48

*Summary of metaanalysis overall analysis, and various sensitivity analyses with k: number of comparisons used; effect size Hedge's g, heterogeneity indicator I^2^; if I^2^ is high the random effects model is indicated; italics document appropriate main model*.

A sensitivity analysis showed that the 16 preventive trials produced an effect size of similar magnitude, which is not significant (*g* = 0.20; *z* = 1.9; *p* = 0.06). The two treatment studies, both of low quality (31, 40), showed no effect (*g* = 0.04; *z* = 0.8; *p* = 0.8) over placebo.

Studies with higher potencies produced a smaller effect size (*g* = 0.18; *z* = 1.81 *p* = 0.07) than the single study with a low potency (g = 0.21), which is, however, not significant.

Interestingly, studies of higher quality, as determined by the Cochrane risk of bias and the quality assessment tool for quantitative studies by Thomas et al., produced a higher effect size, compared with studies of low to moderate quality. The latter (*n* = 10) had a smaller Effect size of g = 0.09, which is not significant and not heterogeneous. High quality studies (*n* = 8) have an effect size of *g* = 0.29, which just misses formal significance (*p* = 0.057).

Metaregressions exploring whether year of study or study size have any influence on effect sizes were not significant, nor was a meta-regression on duration of study (data not shown).

**Figure 3:**
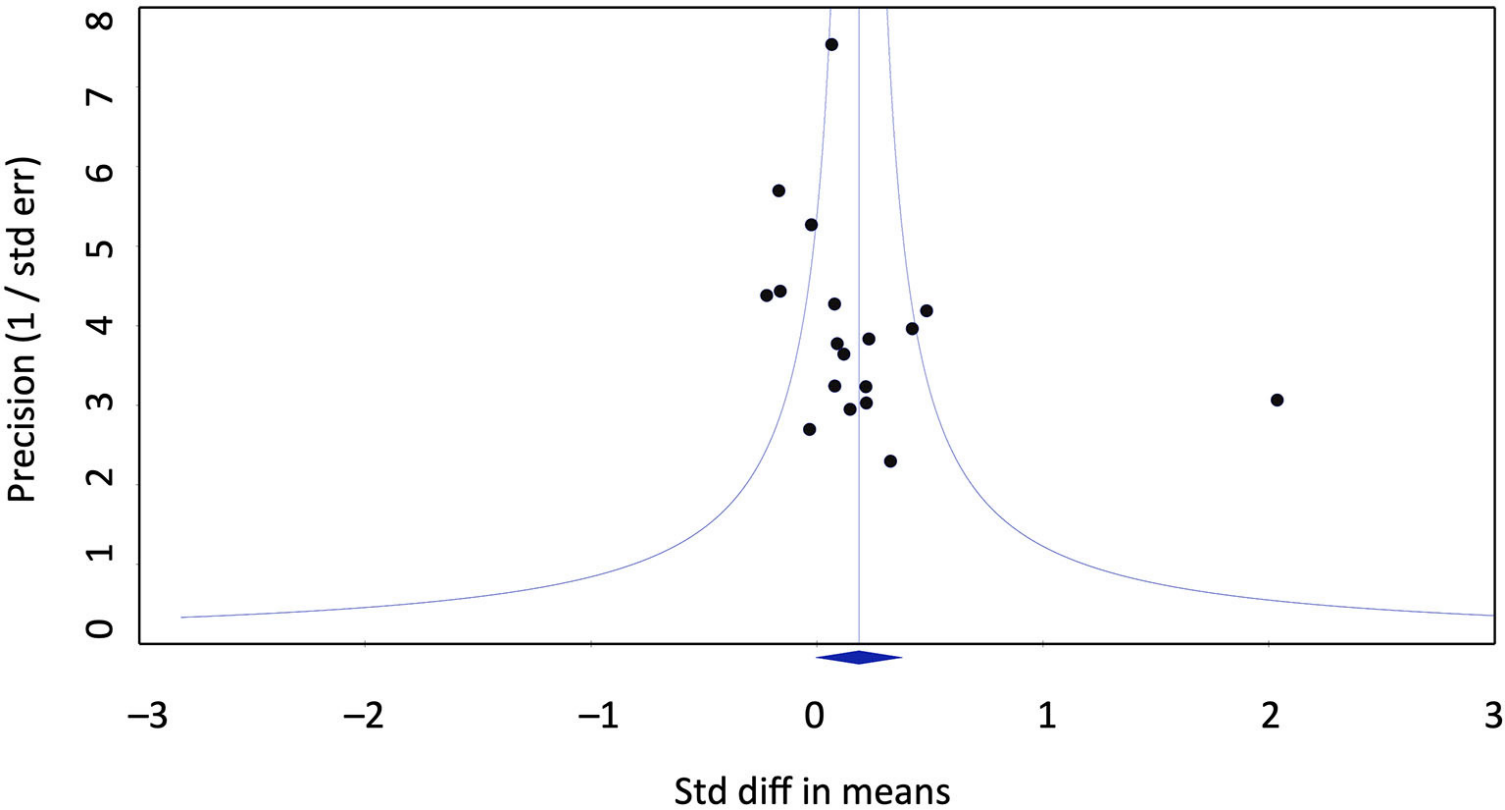
Funnel plot of placebo-controlled trials.

Publication bias does not seem to account for the positive effect of homeopathy vs. placebo. Eggers regression test is not significant (intercept 2.07; confidence interval −0.6/4.77). Duwal and Tweedie's trim and fill method does not impute missing studies to the left of the mean, i.e,. favoring placebo, only to the right of the mean. This is not a realistic assumption as it would mean that seven studies favoring homeopathy over placebo would have gone unpublished, raising the effect size to *g* = 0.39. The funnel plot does not exhibit a distortion (Figure 3).

### Arnica vs. Other-Than-Placebo Comparator

The effect size for *Arnica* vs. comparators other than placebo, most of them active comparator studies, is highly heterogeneous and therefore a random effects model is used. This is non-significant with *g* = 0.28.

We tried to identify the source of this heterogeneity. If the single study, which compared against no-treatment (but presumably allowed conventional pain medication), is removed from the analysis, the overall result remains unchanged (sensitivity analysis 4, Table 2).

The type of comparator is one differentiating factor. The two studies that use standard care as a comparator have a larger and significant effect size, which is homogeneous of g = 0.5 (*p* = 0.03). Since they are at the same time part of the non-randomized study set this is likely the more important moderator (sensitivity analysis 6, Table 2), and non-randomized studies exhibit a significant effect size of *g* = 0.49.

The way how homeopathy is used, preventive or as a formulaic treatment, changes the results (sensitivity analysis 7, Table 2). The preventive use produces a non-significant effect size of *g* = 0.23, therapeutic use a higher effect size of *g* = 0.35, which is, however not significant.

High quality studies produce a higher effect size than lower quality studies (sensitivity analysis 8, Table 2). However, these effect sizes are not significant.

The comparison of potencies does not clarify heterogeneity, as both groups of studies, with high and low potencies, have similar, non-significant effect sizes (high potencies: *g* = 0.15; low potencies: *g* = 0.22; sensitivity analysis 9, Table 2; one study with undeclared potency had a high and significant effect size of *g* = 1.1).

**Figure 4:**
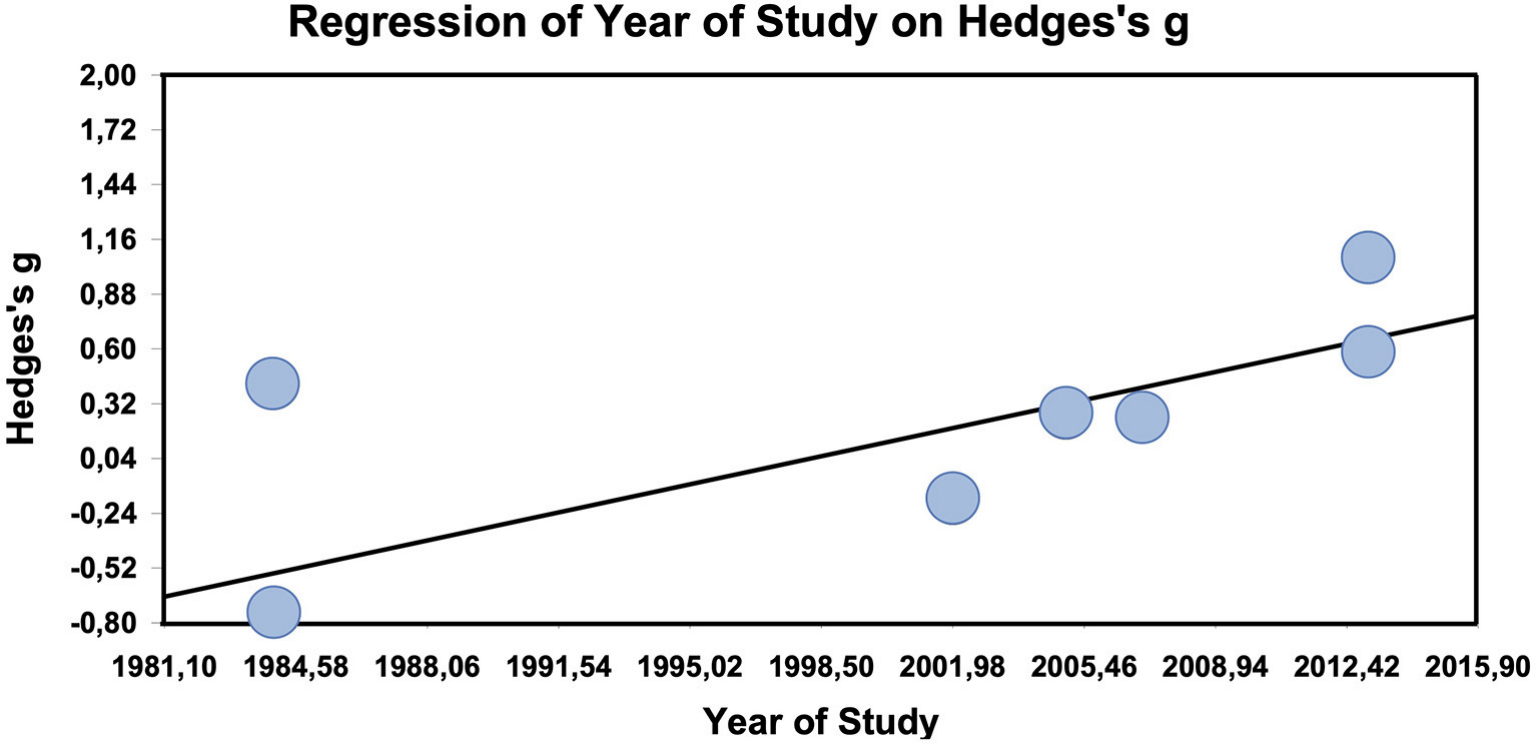
Meta-regression of year of study on effect size in other-than-placebo-controlled trials (*n* = 7).

The metaregression of study year on effect size is significant with a significant slope of 0.03 (*z* = 2.1, *p* = 0.04; maximum likelihood estimation, Figure 4; only clearly designated active comparator trials used, which are at the same as the randomized studies). The metaregression of duration of study on the effect size is not significant (data not shown).

## Discussion

In this metaanalysis of studies testing homeopathic *Arnica* as a medication to prevent and treat postoperative pain, bleeding, swelling, and discomfort after surgery, we found a small effect size of *g* = 0.18 in 18 placebo-controlled trials that missed conventional significance by a small margin, and a non-zero, not significant effect size in active comparator studies, which is likely inflated, because non-randomized studies of lower quality contribute with a large effect size.

In comparison with a recently published systematic review on perioperative Arnica (tinctures and homeopathic potencies), our analysis adds the quantitative cumulative effects of homeopathic Arnica compared the placebo to the descriptive results and summarizes for the first time the clinical effects of homeopathic Arnica compared with an active agent (17).

The outcomes used to document this effect in trials were varied and ranged from pain, which was documented in most of the studies, to swelling, edema, discomfort, or physical measures of range of movement. The studies were most often of smaller surgical interventions, such as carpal tunnel surgery, oral-facial, or dental surgery. The heterogeneity is high in these studies, and clearly limits the results, though it can only be partially explained according to our analysis. Studies that are of better quality have a higher effect size. The findings of sensitivity analyses are in themselves not very robust, as they are based on only a few studies or are not significant. Preventive usage has a lower effect size.

Clinical heterogeneity due to the variety of surgical procedures may have contributed to the wide range of outcome measures. As our analysis model did not separate different types of outcome measures, one might argue that this has conflated effects. This could be true, as effect sizes within studies vary quite a lot. However, since there was no uniform best set of measures that would have been available in all studies, we deemed it better to adopt a conservative strategy of synthesis of all information rather than lose a lot of information for the sake of consistency. Even pain measures varied widely, from real visual analog scale, to nurse-reported ratings, and to area under the curve measures of all pain data. Thus, it would have been possible, but impractical to analyze certain outcome types separately and would likely not have changed the overall result considerably. Thus, our attempt to rather include all information and estimate a robust effect is an analytical decision that might have erred toward the conservative side. If only those studies that used placebo-controls and VAS measures of pain are considered descriptively, then the effect of Arnica can be quantified as lying between a reduction of 5 and 9 mm visual analoque scale (VAS) pain rating.

The active comparator trials produced a small, but non-significant and heterogeneous effect size. In the active comparator trials, the only clear moderator is whether studies were randomized or not. If they were, the effect size converges against zero, i.e., there is no difference between homeopathy and active treatment. Thus, homeopathy seems to work as well as anti-inflammatory substances, which makes sense, as these substances also have an effect over and against placebo (48, 49). Interestingly, the metaregression suggests that in active comparator studies effect sizes against the comparators tend to increase significantly by 0.03 points per year.

The effect size of the overall effect is just missing conventional significance and is small. This lack of formal significance is due to the high heterogeneity of the trials. Sensitivity analyses, see below, can clarify some of this heterogeneity. From a homeopathic point of view, a part of this might be due to the fact that *Arnica* is only partially indicated in surgical wounds, despite its popularity. The established homeopathic indication is blunt trauma, lacerated, internal wounds, and bleeding from such wounds (2–5). Surgical wounds with clear cuts, where tissues have not been “violently stretched” (5) are a different indication, but due to the popularity of *Arnica* and because it seems such an easy indication to study, a lot of studies investigated *Arnica* in surgery without profound homeopathic reasoning. Our analysis shows that it is possible to use *Arnica* effectively in surgery, but not necessarily optimal. The variation in effect sizes is large. In some cases *Arnica* might be counter-indicated, especially if used in a preventive mode, as it might facilitate bleeding (11) and diminish fibrinogen (50). Only two trials were included in the sensitivity analysis of therapeutic use of *Arnica* with placebo-controlled trials. This might explain, why this effect is near-zero and non-significant. The majority of placebo controlled trials were preventive.

It might be worthwhile, therefore, to study other remedies for surgery, such as *Staphisagria* or *Bellis perennis*, either alone or in combination. As we included only pure *Arnica* studies, some studies were not included that used *Arnica* in combination with other remedies, which might have had clinically more interesting results (51–53). We propose to analyze these separately.

The studies that had been conducted so far were rather small. The largest study included 237 patients, and only four studies had more than 100 participants. Considering the small overall effect size, the individual power of studies was small, which is part of the reason why this intervention is debated. However, a metaregression of study size on effect size did not reveal a significant slope, although the slope was slightly negative. Thus, study size in itself does not have any influence on effect size estimation.

Publication bias is an unlikely explanation for our findings, as the analytical methods of publication bias used point out that either an unrealistically large number of studies would have gone unpublished or studies favoring homeopathy were unpublished, none of which is a reasonable assumption.

Active comparator trials compared *Arnica* against standard anti-inflammatories, such as ibuprofen, paracetamol, or diclofenac. The overall effect size is positive but not significant, favoring *Arnica*. This is mainly due to the large effect sizes of non-randomized trials, and hence likely overestimated. However, looking only at the randomized trials presents a non-significant small negative effect size. This suggests that overall Arnica and antiinflammatories are largely comparable.

The effect size is clinically small. *Arnica* in itself might be a suboptimal homeopathic indication for bleeding and swelling after surgery. Thus, an analysis summarizing studies with other remedies or combinations might be warranted. However, considering that *Arnica* is very cheap and an easy intervention, one might consider it worthwhile in cases where patients ask for it. Since the effect size is larger, when used therapeutically, and because of the underlying rationale, we would recommend using it only therapeutically, not preventively.

We conclude that homeopathic *Arnica* produces a small effect size across a series of 18 placebo-controlled trials, which just misses significance, and it is equal in effectiveness to conventional non-steroidal anti-inflammatory drugs in the treatment of postoperative pain, swelling, and functional limitations.

## Data Availability Statement

The data analyzed in this study is subject to the following licenses/restrictions:The data was taken from the results of published clinical studies. The data-set is available from the authors on request. Requests to access these datasets should be directed to Katharina Gaertner, katharina.gaertner@uni-wh.de.

## Ethics Statement

The study was performed according to Swiss law. As a research project analysing using literature data only, the analyses do not require ethical approval. Before any data-analyses were carried out, the project was registered with the international prospective register of systematic review (PROSPERO) on April 28th 2020. All consecutive procedures were logged in a protocol (procedural minutes).

## Author Contributions

Literature search and screening procedures was done by KG. Data extraction and quality assessments were performed by KG and HW. HW executed calculations and drafted large parts of the manuscript. All authors complemented the manuscript and approved the final version.

## Funding

This investigation was funded by Förderverein komplementärmedizinische Forschung, Arlesheim, Switzerland. The funding organization had no role in the design and conduct of the study; collection, management, analysis, and interpretation of the data; preparation, review, or approval of the manuscript; and decision to submit the manuscript for publication.

## Conflict of Interest

KG is a medical doctor with special education in homeopathy. The remaining authors declare that the research was conducted in the absence of any commercial or financial relationships that could be construed as a potential conflict of interest.

## Publisher's Note

All claims expressed in this article are solely those of the authors and do not necessarily represent those of their affiliated organizations, or those of the publisher, the editors and the reviewers. Any product that may be evaluated in this article, or claim that may be made by its manufacturer, is not guaranteed or endorsed by the publisher.
