# Supplementary material for: Is Homeopathic Arnica Effective for Postoperative Recovery? A Meta-analysis of Placebo-Controlled and Active Comparator Trials
Source: Front Surg. 2021 Dec 17;8:680930. doi: 10.3389/fsurg.2021.680930 (PMC8718509; doi:10.3389/fsurg.2021.680930)
Supplement: Supplementary file 1 [file Data_Sheet_1.docx]

# **Supplement 1: eligible studies**

# **Literature search and data selection**

An extensive and comprehensive literature search for an update of evidence from homeopathic intervention studies was carried out upfront (1). The identified records of 639 controlled studies with homeopathic interventions were processed for this review and meta-analysis on Arnica in surgery as follows:

## Study selection process

Data records of the references obtained from the preliminary search were managed by means of the citation manager software Endnote TM Versions X7, X8 & X9 (Clarivate Analytics, Boston, United States). After removal of duplicates and screening by two reviewers, full-texts were retrieved for all reports that seemed to meet inclusion criteria for the literature update^[[1]](#footnote-1)^* or where any uncertainty was present. References were transferred into a standardized and piloted Excel^®^-file, Version 16.16.10 (Microsoft Corporation, Redmont, United States) for further data extraction as described in the study protocol of the literature update (1).

## Data items and data collection process:

Descriptive characteristics of the studies (e.g. study aims, target population, study- and intervention design, interventions and outcomes) were extracted and the studies were allocated to the ICD-10 codes of the respective conditions. Therapeutic and prophylactic studies were processed separately. The prophylactic use of homeopathic medicines was defined as an intervention to which the study population was exposed prior to the exhibition of symptoms or in a symptom-free period.

## Surgical studies

The following ICD-10 categories were considered for pain and wound healing after surgical procedures: Z98.8 Pain during surgical Follow-up (therapeutic use), Z48.8 surgical follow up: hematoma, oedema (therapeutic use), Z48.8 surgical follow up: wound healing (preventive use). Forty-two studies met this criteria, whereas 32 records had Arnica as intervention. These records were potentially eligible for the review, though it was opted to exclude the studies, which investigated Arnica in combination with another homeopathic substance, e.g. Arnica and Hypericum. That way, 23 publications were included in the review.

**References**

1. Gaertner K, Walach H, Baumgartner S, Frass M. Update of empirical evidence: frame-work protocol for the systematic evaluation of homeopathic intervention studies (HOMIS) in humans. Version 1.02020. Available from: <https://zenodo.org/record/4066778#.X3subC2w2-U>.

1. * controlled clinical investigations (RCTs or NRS), employing one or more homeopathically processed substances on humans exhibiting a clinically relevant disease (treatment interventions) or on humans in danger of developing a disease (prophylactic interventions) [↑](#footnote-ref-1)
