# Supplementary material for: Is Homeopathic Arnica Effective for Postoperative Recovery? A Meta-analysis of Placebo-Controlled and Active Comparator Trials
Source: Front Surg. 2021 Dec 17;8:680930. doi: 10.3389/fsurg.2021.680930 (PMC8718509; doi:10.3389/fsurg.2021.680930)
Supplement: Supplementary file 2 [file Data_Sheet_2.docx]

**Arnica-Meta-Analysis**

Started: April 2019; continued March 2020; finished March 12^th^ 2020 with provisional data-base; in between: Prospero registration by Katharina; pausing

Supplement 2 - Extraction Report and Procedures Description

**Harald Walach**

Content data of eligible studies was retrieved from a previously created excel sheet. The method of the preliminary data extraction is available from the protocol and procedural framework strategy for a global evaluation of the evidence body from homeopathic intervention studies (Gaertner, Walach, Baumgartner, & Frass, 2020)

Changes to Original Excel Sheet

A new Excel sheet was created exclusively for Arnica studies. Headers and studies were copied.

The following new columns were added:

- Comment and narrative results column, duration and adverse events, country, specialty, year of study, in the Descriptives Tab
- A column for a third control in the Results Tab

Cochrane Risk of Bias Tool was coded so that a low score means low risk of bias; with three options of coding

After review (cross-check of extracted data by KG) were added:

- In the descriptives tab: Intervention flexibility column
- In the results tab: review column “checked”
- In the Cochrane and the model validity tab: second assessment columns “KG”
- In the external validity tab: judgement columns were renamed “KG”

Changes or queries were highlighted yellow and were resolved by discussion.

Conventions and Procedures

We took ITT or PP data according to the study reports, and preferred ITT if both were reported.

If primary outcome is given, extract primary outcome, ignore the rest or extract rest but mark primary.

If no primary outcome is given, average similar outcomes across time-points, calculate effect sizes per outcome and average across effect sizes

If groups of variables of different domains are given, average across domains first, then average domains

Extract averaged values into Excel sheet; however, this never occurred.

All coding such that positive sign means an ES towards homeopathy better

All studies that contained more than one arm extracted as two studies.

Standard deviations derived from standard errors of the mean by the formula SD = SEM*SQRT(n), or by the Cochrane calculator, if only p-values were known.

Odds ratio (1 case) converted into d following the forula d = sqrt(3)/pi(logOR). SDs for difference calculated by spreadsheet formula sd_diff_ = sqrt(sd_1_^2^ + sd_2_^2^). Currently as many as 4 effect-sizes/outcomes extracted, as this was the maximum number. If more than one outcome given and no primary outcome mentioned, I extracted all and averaged them equally.

Here is a description of how we dealt with various problems.

**Camacho et al**

This study provides only statistics of a Kruskall Wallis test across three groups and not enough data for extraction of any effect size. Hence it was ignored.

Second extraction/quality review: not assessed

**Jeffrey**

The VAS-score and associated SD had to be extracted from the graph, via visual approximation

Second extraction/quality review: sample size is unclear (Text and table are contradictory: Arnica group had 20? patients but three patients in BOTH groups had the other surgery??), attrition rate is 0 for PP-Analysis: do we always use PP-analyses?? Data checked. Cochrane: Selection bias is rated 0=random sequence generation, but judgement is rated 1=unclear 🡪 why?; other differences to discuss

Query solved by discussion: we used the PP values as reported. Judgement for the Cochrane quality assessment was corrected to 0.

**Karow et al**

Since this is a study based on a hierarchical non-parametric procedure and an equivalence trial it has to be dealt with separately; hence marked green in spreadsheet

The median values have been used as conservative estimates for means; averaged over days. SDs have been estimated from the p-values: p-values used to determine t-scores (at this sample size approximately identical to z-scores). t-scores used to estimate SEmean. Assuming similar variances SEmean/2*SQRn1 yields SD estimate (SQRn1 is 44). The estimates of the SDs is thus comparatively shaky, but also conservative.

Second extraction/quality review: data not checked (except sample and group size and correctness of formula for the calculations). HPHPP global rating of HW should be moderate (2), when confounders are weak (3) 🡪 changed

**Robertson**

straightforward, except SDs for the secondary outcomes calculated as sums of single SDs, according to the general sum of means used; but as a primary outcome is given, this might be not used anyway

Second extraction/quality review: corrected ITT control-sample from 87 to 97; checked primary outcome; pooled ES was used

HPHPP global rating of HW should be weak (3), when confounders and attrition are weak (3) 🡪 changed

Answer to query: Pooled effect size was used

**Fernandez**

Chi-Square test with three categories reduced to a Chi-Square test with 2 categories, using the Chi-Square calculator at <https://www.socscistatistics.com/> (access 4/4/19); results in same Chi-Square value, but reduced for Yates correction for continuity (i.e. small cell frequency), hence more conservative. This conservative value was used to transform it into d using <https://www.psychometrica.de/effektstaerke.html>, which implements formulae from Rosenthal and Di Matteo

Second extraction/quality review: data not checked (except sample and group size and correctness of formula for the calculations). HPHPP global rating of HW should be weak (3), when design, blinding and confounders are weak (3) 🡪 changed

**Gonzalez**

is very similar to Fernandez; same procedure

Second extraction/quality review: data not checked (except sample and group size and correctness of formula for the calculations). HPHPP global rating of HW should be weak (3), when design, blinding and confounders are weak (3) 🡪 changed

**Totonchi**

Tree-armed with only means and p-values; 3 variables measured at 2 time points, in addition also difference between time point 2days and 8days; I take only the 8 day values from three groups, as it seems the most straightforward; the analysis applied was ANOVA, but no post-hoc test results or SDs are given; hence the SD has to be estimated. I used the grand mean as an orientation for an estimator for the SDs and in the end used either the grand mean or 2*the mean, whichever was more plausible.

Second extraction/quality review: data checked. Pooled ES is missing, primary outcome used for MA?. HPHPP global rating of HW should be weak (3), when confounders and drop-outs are weak (3) 🡪 changed

Answer to query: Pooled effect size calculated and used for meta-analysis

**Brinkhaus**

Three trials, entered, coded and analysed separately. As time point the last was used.

Second extraction/quality review: data for primary outcome checked. Attrition rate % is unclear to me…

Answer to query: attrition rate checked and corrected

**Chaiet**

Badly reported. The sds were recovered from t-values applied to the exact p-values reported, always for the last time point (day 9) and for both outcomes, the other time points were ignored.

Second extraction/quality review: data checked except recovered sds. HPHPP global rating of HW should be moderate (2), when confounders are weak (3) 🡪 changed

**Puerta Horta**

Reported dichotomized values of VAS-scales into satisfactory relief and non-satisfactory relief for Arnica vs. conventional. Odds-ratios from 2*2 table (conservative estimate by replacing 0 with 1 in order to be able to regain a numerical value for OR); then transformation via d = sqrt3/pi (logOR).

Second extraction/quality review: data not checked (except sample and group size and correctness of formula for the calculations).

**Hart et al**

Reported non-parametric data and results from a linear model (adjusted means). I took the adjusted means, calculated the SDs from the SE of the difference via the Revman tool and used the four parameters presented (VAS pain, time, area under the curve and peak) and averaged them

Second extraction/quality review: data checked except calculated sds.

**Kaziro**

This is a badly presented trial. The VAS was in fact a four-stage numerical scale and only percentages are given in a hand-drawn graph. I recalculated the original data from those percentages, using approximations and plausibility checks on day 8, because this was the date the stats had been calculated for. Same for edema. I ignored the variable “wound breakdown” because this was given in a bar graph as number of 10%, which was unclear so did not lend itself to recalculation. Outcome 3 was mout opening in mm, derived from the graph on day 8.

Second extraction/quality review: data not checked (except sample and group size and correctness of formula for the calculations).

**Kotlus**

This trial is a well conducted intraindividually controlled study. However, since no primary outcome was defined, all outcomes were extracted and averaged. Data as reported in the text

Second extraction/quality review: data checked, p-value changed to 0.85, because if rounded it would be 0.9

**Lotan et al**

Clearly defined primary outcome (time to removal of drain) which is clearcut and continuous. Although there were differences in weight of breasts removed the stats did not adjust for that, which is a pity, and the data for the opioids was not mentioned and forgotten in the table, and the referees obviously overlooked this. But as the data were straightforward I extracted only the primary outcome.

Second extraction/quality review: ignored because intervention was Arnica and Bellis

**Macedo et al**

This was an obviously well conducted but badly reported trial. The data were extracted from the figures, by blowing up the PDF to match the ruler and then measured by ruler on the screen (edema, mouth opening, pain). The same goes for the SEMs, which the error bars very likely were. As it is a intraindividually controlled trial I used SQRT(32) as the multiplicator for the SEMs to derive the SDs. I did not use number of tablets, because the time to escape medication was given as a fourth numerical value with precision. However, what is reported as a SD in the publication is a SEM and hence this value has been converted to SDs in the spreadsheet and all 4 outcomes were used and averaged.

Second extraction/quality review: data not checked (except sample and group size and correctness of formula for the calculations), because when I look at the graphs, it is uclear to me which outcome is which.

**Pinsent et al**

This study is badly reported, as the number of dropouts is not clear and the final number not legible in the PDF. I reconstructed it, assuming equal numbers of dropouts. The SDs have been back-calculated from the difference and the p-values

Second extraction/quality review: data checked and calculations are correct, though sample and group size size is unclear. Attrition rate corrected according to the group size used by HW…however, authors report 72 valid CRFs, hence evaluated subjects are probably 36/36 (if distributed equally)???

Outcome 1_mean corrected according to text (typo). HPHPP global rating of HW should be weak (3), when selection and drop outs are weak (3) 🡪 changed

Query solved by discussion: Pooled ES was used for meta-analysis

**Pöllmann & Hildebrandt**

This is a non-randomised 3-armed study, with one study arm (n = 7) comprising of patients with conventional medication, one arm with homeopathy, and one arm with no treatment. I divided it in two studies. The mean and SEMs were taken from the graphs, but graph 2 and 3 are obviously wrongly placed and the legend of graph 2 belongs to graph 3 and vice versa.

Second extraction/quality review: data not checked (except sample and group size and correctness of formula for the calculations). Active treatment and no treatment group size needs to be inversed. How were SDs calculated?

Answer to query: SEMs were derived from the graphs and SDs calculated.

**Ramelet**

This study is well conducted in terms of trial methodology. The outcome – hematoma – was unfortunately only reported as a 3-point rating and evaluated via chi-square. I have converted the data back into continuous data and calculated means and sds and have used these for further analysis.

Second extraction/quality review: data not checked (except sample and group size size and correctness of formula for the calculations).

**Seeley et al**

This study did not use any predefined outcome. So I used the data from the graph and measured it with a ruler on day 13; the second outcome is the one reported in the text, days after people were comfortable going out, which is reported with mean and SD, and the other two outcomes are the colour measurements which were the objective data. The mean values are given and the SDs were back-calculated using the Revman calculation tool.

Second extraction/quality review: reported values checked. It was unclear which day was used for the outcome. From what I see at the graph day 13? HPHPP global rating of HW should be moderate (2), when confounders are weak (3) 🡪 changed

Answer to query: Day 13

**Sorrentino**

Second extraction/quality review: ITT-values used and checked. Attrition rate corrected. This study was not reported in the procedural minutes…

Report of methods: There was nothing to report, as all data were clearly available from the publication.

**Souza et al.**

This Brazilian study is actually well performed, from what one can tell, but badly reported. They report the data for three categories in a graph of swelling on the target day 3 in three measured segments of the mouth to head opening. I reconverted these data as categorical ones in the spreadsheet and calculated means and Sds which I then used for further analysis in the spreadsheet.

Second extraction/quality review: data not checked (except sample and group size and correctness of formula for the calculations), as data seems to be taken from the graph

**Stevinson et al**

This is a three-armed study which has been extracted as two different arms, Arnica C6 and Arnica C30 vs. the same placebo group. Although well planned and reported, the study is missing decisive information in its report and is also using the wrong statistical measure. There are medians and Chi-Square stats over the three groups. In addition, full data-ranges are given, no interquartile range. This cannot be used to recalculate a SD. But the authors indicated data-categories for ratings by clinicians. These I used for deriving SDs for this particular data-set and used these for the meta-analysis on day 4. I used median as mean scores and used a conservative interpolation of SD = mean for the two main criteria. I adopted this procedure also for the colour data

Second extraction/quality review: reported values checked.

**Wolf et al 2002**

This study is not well reported. The baseline data give the circumference of the femur as mean and SD scores, and the graphs give percentage change from baseline. So I used these scores to calculate the scores at the end of treatment. The other parameters are given as non-significant differences, but with no numerical scores to go with it was impossible to extract further information. So I coul only use this single outcome parameter.

Second extraction/quality review: data checked. However, to my mind the graph shows less than 1% increase of upper leg circumference for the Arnica-group and the values would be different…

Query discussed and corrected: That is true, but in order to reach the full difference effect, first the swelling on day 0 had to be added to the baseline-circumference before the difference could be calculated.

**Wolf et al 2003**

This is a well reported study. I used the two outcomes size of the change of the area of the hematoma, measured as change from baseline, and the pain-change as reported in the text including the appropriate SDs.

Second extraction/quality review: data checked. However, to my mind it would be more reasonable to use the values for reduction of haematoma as baseline-haematoma after surgery was not equal…

Query discussed: values used as reported

**Erkan 2019**

This study is badly reported. Neither is clear which ones the important outcomes are, nor are the data presented well. The first data presented are the physicians’ evaluation which are presented as a 3-categorial table. I converted that into a continuous measure recalculating mean and SDs. Of the patient data I used only the first two, pain and swelling, as these are the ones that are also comparable to other studies. The other four variables (phonetics, daily chores, eating, slepping) I did not use. I converted the frequencies into rating scale data and caculated means and sds. Also, it is unclear how these variables were coded or evaluated (what would it mean to say “sleeping – never, mild, moderate, severe”? Would it mean sleeping difficulties, or what else?). Therefore they were not considered further.

Second extraction/quality review: data not checked (except sample and group size and correctness of formula for the calculations), as outcome was completely unclear to me. Erkan: HPHPP global rating of HW should be moderate (2), when only data collection is weak (3) 🡪 changed

**Quality assessments**

All studies were assessed independently by two reviewers (HW and KG) with the following assessment tools:

- Cochrane Risk-of-bias 1.0 for randomized controlled trials
- the quality assessment tool for quantitative studies by the Effective Public Health Practice Project (EPHPP) as the latter allows quality assessment for non-randomized studies
- The external validity questions by Black and Downs

In all cases agreement was found by discussion, hence no further agreement statistics was necessary..

For the assessment of Model validity three criteria were applied:

- If the intervention of the study was preventive or therapeutic (a preventive approach was assumed, if the application of the intervention drug Arnica tarted before the operation), whereas a preventive intervention was judged as unclear model validity
- If the intervention drug Arnica was diluted below Avogadros number or above (a dilution below Avagadros number was set for the potencies D1-D7 and C1-C4 for all substances), whereas low dilutions with unfrequent repetitions and high dilution with very frequent repetitions were judged as low model valitidy
- If the intervention was flexible concerning the frequency of application or not, whereas a flexible approach was judged as high model validity and an inflexible approach either as unclear or low model validity (depending on the potency)

5.4.20

We discussed all discrepancies and agreed on common definitions, solved all questions and saved the new data in the file Arnica_Daten_Katharina_2 which is the file for calculating the meta-analysis.

**Reference**

Gaertner, K., Walach, H., Baumgartner, S., & Frass, M. (2020). Update of empirical evidence: frame-work protocol for the systematic evaluation of homeopathic intervention studies (HOMIS) in humans. Version 1.0. doi:10.5281/zenodo.4066778
