# Supplementary material for: Is Homeopathic Arnica Effective for Postoperative Recovery? A Meta-analysis of Placebo-Controlled and Active Comparator Trials
Source: Front Surg. 2021 Dec 17;8:680930. doi: 10.3389/fsurg.2021.680930 (PMC8718509; doi:10.3389/fsurg.2021.680930)
Supplement: Supplementary file 3 [file Data_Sheet_3.docx]

| Supplement 3 - Arnica in surgery – characteristics of excluded studies | | | | | | | | |
| --- | --- | --- | --- | --- | --- | --- | --- | --- |
| ICD-10 category | **Author** | **Year** | **Condition/ pathology** | **Publication status** | **Study design** | **Intervention** | **Comparators** | **Reason for exclusion** |
| Z48.8 surgical follow up: wound healing (preventive use) | Cornu | 2010 | Aortic valve surgery | peer-review | RCT | Arnica 5C & Bryonia 5C | Placebo | Arnica in combination |
|  | Cummerow | 2006 | Dermatologic excisions | thesis | RCT | metaF-R201^1^ | Placebo | Intervention not Arnica |
|  | Florit | 2001 | Dental extractions | non peer-review | NRS | Traumeel S^2^ | standard analgesics | Intervention not Arnica |
|  | Lotan | 2020 | Mastectomy in Breast Cancer | peer-review | RCT | Arnica montana C30 & Bellis perennis C30 | Placebo | Arnica in combination |
|  | Machado Ramos | 2016 | Dental extractions | non peer-review | NRS | Hypericum C200 | standard analgesics | Intervention not Arnica |
|  |  |  |  |  |  | Hypericum M |  |  |
|  | Michaud | 1981 | Maxillo-facial surgery | thesis | RCT | Arnica C15 & Apis C7 | Placebo | Arnica in combination |
|  | Paris | 2008 | Cruciate ligament reconstruction | peer-review | RCT | Complex homeopathic formula^3^ | Placebo | Intervention not Arnica |
|  | Rafai | 2005 | Dental extractions | thesis | RCT | Arnica C30 & Hypericum D30 | Placebo | Arnica in combination |
|  | Rodríguez Gutiérrez | 2008 | Dental extractions | peer-review | RCT | Arnica C200 & Hypericum C200 | standard analgesics | Arnica in combination |
| Z48.8 surgical follow up: hematoma, edema | Tena | 2009 | Traumatic hyphema | non peer-review | RCT | Arnica C6 & Phosphorus C30 | standard care | Arnica in combination |
|  | Tan Suárez | 2008 | Dental surgery | peer-review | NRS | Calendula C30 | Alvogyl | Intervention not Arnica |
| Z98.8 Pain during surgical Follow-up | Bendre | 1980 | Dental extractions | non peer-review | RCT | Arnica & Hypericum C200 | Placebo | Arnica in combination |
|  | Camacho | 2008 | Dental extractions | conference proceedings | NRS | Arnica C7 | Naproxen 550mg | Data not sufficient |
|  |  |  |  |  |  |  | Placebo |  |
|  | Donati | 2015 | Dental surgeries | non peer-review | RCT | Arnica D3 & Silicea Comp^4^ | Naproxen 500mg & Amoxicillin | Arnica in combination |
|  | Lökken | 1995 | Dental extractions | peer-review | RCT | Individualized homeopathy | Placebo | Intervention not Arnica |
|  | Mazzocchi | 2012 | Dental surgeries | peer-review | NRS | Symphytum C5 | standard care | Intervention not Arnica |
|  | Singer | 2007 | Halux valgus surgery | peer-review | RCT | Traumeel S^2^ inj. only | standard care | Intervention not Arnica |
|  |  |  |  |  |  | Traumeel S^2^ inj.+p.o. |  |  |
|  | Singer | 2010 | Halux valgus surgery | peer-review | RCT | Traumeel S^2^ | Placebo | Intervention not Arnica |
|  | Taylor | 2015 | Circumcision | thesis | RCT | Staphysagria C30 | standard care | Intervention not Arnica |
|  | Vantour | 2017 | Dental extractions | non peer-review | NRS | Arnica C9 & Hypericum C9 | standard care | Arnica in combination |

Capture: NRS=non randomised study; RCT=randomised controlled trial; ^1^ Components: Calendula officinalis D2, Delphinium staphisagria D6, Graphites D12, Thiosinamin D2; ^2^ Components: Arnica montana D2, Calendula officinalis, D2, Hamamelis virginiana D2, Achillea millefolium D3, Atropa belladonna D4, Aconitum napellus D3, Mercurius solubilis Hahnemanni D8, Hepar sulfuris D8, Chamomilla recutita D3, Symphytum officinale D8, Bellis perennis D2, Echinacea angustifolia D2, Echinacea purpurea D2, Hypericum perforatum D2; ^3^ Components: Arnica montana C5, Bryonia alba C5, Hypericum perforatum C5, Ruta graveolens D3; ^4^ Components: Argentum nitricum D20, Atropa belladonna, D14, Quarz D21
