# Supplementary material for: Is Homeopathic Arnica Effective for Postoperative Recovery? A Meta-analysis of Placebo-Controlled and Active Comparator Trials
Source: Front Surg. 2021 Dec 17;8:680930. doi: 10.3389/fsurg.2021.680930 (PMC8718509; doi:10.3389/fsurg.2021.680930)
Supplement: Supplementary file 4 [file Data_Sheet_4.docx]

## **Supplement 4 – Lists of studies**

## List of included studies

1. Brinkhaus B, Wilkens JM, Ludtke R, Hunger J, Witt CM, Willich SN. Homeopathic arnica therapy in patients receiving knee surgery: results of three randomised double-blind trials. Complement Ther Med. 2006;14(4):237-46.

2. Chaiet SR, Marcus BC. Perioperative Arnica montana for reduction of ecchymosis in rhinoplasty surgery. Annals of plastic surgery. 2016;76(5):477-82.

3. Erkan E, Parpar K, Develi T, Gündoğar M, Gürler G. The efficacy of homeopathic Arnica montana 200 CH on dental surgical treatments: a double-blind, placebo-controlled study. The European Research Journal. 2018.

4. González Sánchez AM GHJ, Rivaflecha GF, Morales Alcolea Y. Efectividad de remedios homeopáticos en niños operados de estrabismo. Medisan. 2014;18(10).

5. Hart O MM, Lewith G, Miller J. Double-blind, placebo-controlled, randomized clinical trial of homoeopathic arnica C30 for pain and infection after total abdominal hysterectomy. J R Soc Med. 1997;90:73-8.

6. Jeffrey S BH. Use of Arnica to relieve pain after carpal-tunnel release surgery. Altern Ther Health Med. 2002;8(2):66-8.

7. Karow JH, Abt HP, Frohling M, Ackermann H. Efficacy of Arnica montana D4 for healing of wounds after Hallux valgus surgery compared to diclofenac. J Altern Complement Med. 2008;14(1):17-25.

8. Kaziro GSN. Metronidazol (Fagyl) and arnica montana in the prevention of post-surgical complications, a comparative placebo controlled clinical trial. Br J Oral Maxillofac Surg. 1984;22:42-9.

9. Kotlus BS, Heringer DM, Dryden RM. Evaluation of homeopathic Arnica montana for ecchymosis after upper blepharoplasty: a placebo-controlled, randomized, double-blind study. Ophthal Plast Reconstr Surg. 2010;26(6):395-7.

10. Macedo S CJ, Ferreira L, Dos Santos-Pinto R. Effect of Arnica montana 6 cH on edema, mouth opening and pain in patients submitted to extraction of impacted third molars. Ärztezeitschrift für Naturheilverfahren. 2005;46(6):381-7.

11. Pinsent RJFHB, G.P.I.; Ives, G.; Davey, R.W.; Jonas, S. Does Arnica reduce pain and bleeding after dental extractions? Brit Hom Res Group Communications. 1986;15:3-11.

12. Pöllmann L HG. Zur Gabe von Arnika, Planta tota D3, bei kieferchirurgischen Eingriffen. Erfahrungsheilkunde. 1993;7:503-7.

13. Puerto Huerta MdCI, L.; Cañete Villafranca, C.R.; . Arnica montana en el tratamiento del dolor después de la odontectomía de terceros molares retenidos. Medisan. 2015;19(5):619.

14. Ramelet A BG, Lorenz P, Imfeld M. Homoeopathic Arnica in Postoperative Haematomas: A Double-Blind Study. Dermatology. 200;201:347–8.

15. Robertson A, Suryanarayanan R, Banerjee A. Homeopathic Arnica montana for post-tonsillectomy analgesia: a randomised placebo control trial. Homeopathy. 2007;96(1):17-21.

16. Seeley B DA, Ahn M, Maas C. Effect of Homeopathic Arnica montana on Bruising in Face-lifts: Results of a Randomized, Double-blind, Placebo-Controlled Clinical Trial. Arch Facial Plast Surg. 2006;8:54-9.

17. Sorrentino L, Piraneo S, Riggio E, Basilico S, Sartani A, Bossi D, et al. Is there a role for homeopathy in breast cancer surgery? A first randomized clinical trial on treatment with Arnica montana to reduce post-operative seroma and bleeding in patients undergoing total mastectomy. J Intercult Ethnopharmacol. 2017;6(1):1-8.

18. Souza LM. Ação Anti-Edematosa: Arnica montana 6ch X Diclofenaco de Sódio 50 mg. Pesquisa Brasileira em Odontopediatria e Clínica Integrada. 2011;11(4):491-6.

19. Stevinson C DV, Fountain-Barber A, Hawkins S, Ernst E. Homeopathic arnica for prevention of pain and bruising: randomized placebo-controlled trial in hand surgery. J R Soc Med. 2003;96:60-5.

20. Totonchi A, Guyuron B. A randomized, controlled comparison between arnica and steroids in the management of postrhinoplasty ecchymosis and edema. Plast Reconstr Surg. 2007;120(1):271-4.

21. Wolf M LR, Rose O. Adjuvante Arnica-Medikation zur schwellungs- und schmerzreduzierenden Wirkung bei operativ versorgten hüftgelenksnahen Frakturen: Kontrollierte, nicht randomisierte Therapiestudie. AHZ. 2002;247(4):141-4.

22. Wolf M TC, Mayer W, Heger M. Wirksamkeit von Arnica bei Varizenoperation: Ergebnisse einer randomisierten, doppelblinden, Placebo-kontrollierten Pilot-Studie. Forsch Komplementarmed Klass Naturheilkd. 2003;10:242–7.

## List of excluded studies

1. Bendre V DS. Arnica montana and hypericum in dental practice The Hahnemannian Gleanings. 1980;47:70-2.

2. Camacho C LS, Melo M, Pedraza C, Vanegas S, Benítez G, Palencia R, Revelo I, editor Effectiveness of homeopathic medicine Arnica 7CH versus Naproxen on post operative extraction of third molar including pain relief. 63rd Congress of the Liga Medicorum Homoeopathica Internationalis; 2008; Oostende, Belgium: Liga Medicorum Homoeopathica Internationalis.

3. Cornu C, Joseph P, Gaillard S, Bauer C, Vedrinne C, Bissery A, et al. No effect of a homoeopathic combination of Arnica montana and Bryonia alba on bleeding, inflammation, and ischaemia after aortic valve surgery. Br J Clin Pharmacol. 2010;69(2):136-42.

4. Cummerow R. Doppelblinde, plazebokontrollierte, randomisierte Phase-III-Studie zur Wirksamkeit und Verträglichkeit des homöopathischen Kombinationspräparates metaF-R201 Tropfen in der peri- und postoperativen Wundbehandlung. Freiburg i.Br., Germany: Albert- Ludwigs- Universität; 2006.

5. Donati G AM, Ghezzi M, Rania V, Luraghi C, Liverani A, Quattrone G, Alivia M, Sparaco AP. Antibiotika/NSAID versus Arnica, Planta tota Flüssige Verdünnung (D3) und Silicea comp., Clobuli velati bei zahnchirurgischen Eingriffen: ein klinischer Vergleich. Der Merkurstab. 2015;69(2):154-7.

6. Florit JR. Efectos de Traumeel S sobre el dolor, inflamación y hemorrhagia postextracciones dentales. La Med Biol. 2001;14(1):18-20.

7. Lökken P SP, Tveiten D, Skjelbred P, Borchgrevink F. Effect of homoeopathy on pain and other events after acute trauma: placebo controlled trial with bilateral oral surgery BMJ. 1995;310:1439-41.

8. Lotan AM, Gronovich Y, Lysy I, Binenboym R, Eizenman N, Stuchiner B, et al. Arnica montana and Bellis perennis for seroma reduction following mastectomy and immediate breast reconstruction: randomized, double-blind, placebo- controlled trial. European Journal of Plastic Surgery. 2020.

9. Machado Ramos SRM, O.; González Docando, Y.E.; Pérez, D.D.; Guerra López, J.R.; Ávila García, M. El Hypericum perforatum como anestésico local en las extracciones dentarias. Mediciego. 2016;22(1):31-6.

10. Mazzocchi AM, A. Observational study of the use of Symphytum 5CH in the management of pain and swelling after dental implant surgery. Homeopathy. 2012;101:211-6.

11. Michaud J. Action d`Apis mellifica et d`Arnica montana dans la prévention des oedèmes post-opératoires en chirurgie maxillo-faciale à propos d`une expérimentation clinique sur 60 observations. Nantes, France: Université de Nantes; 1981.

12. Paris A, Gonnet N, Chaussard C, Belon P, Rocourt F, Saragaglia D, et al. Effect of homeopathy on analgesic intake following knee ligament reconstruction: a phase III monocentre randomized placebo controlled study. Br J Clin Pharmacol. 2008;65(2):180-7.

13. Rafai N. Arnica montana und Hypericum perforatum D30 nach operativer Weisheitszahnentfernung. Aachen, Germany: Rheinisch-Westfälische Technische Hochschule Aachen; 2005.

14. Rodríguez Gutiérrez GMTS, N.; Landrián Díaz, C.I.; Arias Herrera, S.R. Eficacia de la terapia homeopática contra tratamiento convencional en extracciones de dientes temporales. Revista Archivo médico de Camaguey. 2008;12(1):1-9.

15. Singer SR, Amit-Kohn M, Weiss S, Rosenblum J, Lukasiewicz E, Itzchaki M, et al. Efficacy of a homeopathic preparation in control of post-operative pain—A pilot clinical trial. Acute Pain. 2007;9(1):7-12.

16. Singer SR, Amit-Kohn M, Weiss S, Rosenblum J, Maoz G, Samuels N, et al. Traumeel S for pain relief following hallux valgus surgery: a randomized controlled trial. BMC Clin Pharmacol. 2010;10:9.

17. Tan Suárez NHG, C.R.; Tan Suárez, N.T.; Rodríguez Gutiérrez, G.M.; Fernández Carmenate, N. . Tratamiento homeopático vs alvogyl en la alveolitis dental. Archivo médico de Camaguey. 2008;12(3).

18. Taylor EC. Efficacy of a topical application of a homoeopathic protocol as an adjunct to the standard care of the post-surgical effects of circumcision. Durban, South Africa: Durban University of Technology; 2015.

19. Tena SIPA, A.A.C.; González, H.C.; Sánchez, C.P. de; Medellín, O.; González, E.R. . Uso del árnica y el phosphorus en el tratamiento del hifema traumático. Revista Médica de Homeopatía. 2009;2(3):151-5.

20. Vantour ACLP, K.Q.; Filiu, M.M.; Colome, M.E.G.; Luna, A.M.Q. . Efectividad del tratamiento homeopático en extracciones complicadas o laboriosas. Medisan. 2017;21(10):3032-7.
